# A Methodological Assessment of Pharmacist Therapeutic Intervention Documentation (TID) in a Single Tertiary Care Hospital in Jeddah, Kingdom of Saudi Arabia

**DOI:** 10.3390/pharmacy9020097

**Published:** 2021-04-28

**Authors:** Ali F. Alwadie, Anjum Naeem, Meaad A. Almazmomi, Meshail A. Baswaid, Yahya A. Alzahrani, Abdullah M. Alzahrani

**Affiliations:** 1 Pharmaceutical Care Department, Ministry of National Guard—Health Affairs, Jeddah 22384, Saudi Arabia; wadeial@ngha.med.sa (A.F.A.); AnsariAN@ngha.med.sa (A.N.); Almazmomime@ngha.med.sa (M.A.A.); Baswaidma@ngha.med.sa (M.A.B.); 2 King Abdullah International Medical Research Center, Jeddah 22384, Saudi Arabia; 3 King Saud bin Abdulaziz University for Health Sciences, Jeddah 22384, Saudi Arabia; 4 Department of Pharmacy, East Jeddah Hospital, Ministry of Health, Jeddah 22253, Saudi Arabia

**Keywords:** pharmacist intervention, prescribing order entry errors, therapeutic intervention fatigue

## Abstract

Pharmacist intervention has valuable input to the healthcare system by reducing medication errors, costs of treatment and improving therapeutic outcomes. This study aimed to analyze pharmacists’ interventions during the verification of computerized physician order entry and to determine the association between prescribers’ level and type of prescribing errors. In this cross-sectional, observational study, data collection was carried out over three months starting from 1 January 2020 to 31 March 2020. Included were 2405 interventions documented by 52 different pharmacists. The prevalence of prescribing order entry errors was 9.1%. The most identifiable type of intervention was incorrect dilution (40.2%) followed by dose substitution (27.7%). The drug category associated with a high percentage of interventions was perfusion solutions (41%), followed by antibacterial (35%). The number of junior physician orders that required pharmacist intervention was higher than other prescribers (45.2%), followed by specialist and senior physicians, (31.4% and 15.5%, respectively). Prescriber ordering time and types of prescribing errors were shown to have a significant (*p* < 0.05) association. Internal medicine physicians entered the highest percentage of prescribing errors, representing 22.7%. The current study concluded that TID has significant potential to reduce drug-related problems; TID fatigue is a real problem that might be under-reported and addressing this point in future studies would be of great value.

## 1. Introduction

Pharmacist interventions play an important role in measuring and minimizing physician’s prescribing errors [1]. Furthermore, pharmacist interventions have always been seen as valuable input by the patient care community to reducing medication errors, streamlining treatment and reducing treatment costs [2]. The Joint Commission on Accreditation of Healthcare Organizations (JCAHO) has recommended that pharmacists must review all prescriptions before dispensing and has stressed that the outcomes should be documented as a result of direct patient care by the pharmacy [3].

Computerized physician order entry (CPOE) is a technology that allows physicians to enter orders into a computer instead of handwriting them. One of the CPOE benefits is promoting standardization of the drug entry through electronic prescribing of a clear and complete order, leading to a decrease of potential prescribing errors. Studies have documented that CPOE can decrease costs and decrease medication errors [4,5]. Once the physician order entry is completed through CPOE, the pharmacist reviews every order, interprets all medications and resolves all actual and potential order issues before verifying the drug in the patient profile and dispensing [6,7].

On the other hand, medication error remains common and is responsible for significant morbidity and mortality [8]. These errors are often known as adverse drug events (ADEs) and are a leading cause of iatrogenic injury [9,10]. Prescribing errors are the most common ADE and are reported to harm at least 1.5 million people every year [11]. The reports have shown that between 44,000 and 98,000 people in the United States die from medical errors per year [9,12]. Additional medical costs for treating ADEs are also estimated to be $3.5 billion a year without considering loss of wages or other medical expenses [9,13].

In academic and tertiary hospitals, resident physicians place most orders for their patients. Therefore, the majority of medication errors occur at the ordering stage. Little is known regarding the relationship between resident training level and frequency and types of medication order error occurrence. Most studies that investigated residents’ prescribing errors have focused on a variable such as the number of working hours, sleep deprivation and depression [12]. One study investigated the frequency, type of error and severity of written prescription errors in a pediatric emergency department (ED) with regard to resident prescriber specialty and training level. The authors concluded that prescription errors in ED are very common, with an error rate of 59% [14]. Another study checked all inpatient prescription orders issued by residents of internal medicine at an academic medical center from July 2011 to June 2015. Prescribing errors were calculated by pharmacists reporting the error via electronic medical records during real-time order monitoring. The associations between resident training level (postgraduate year [PGY]), timing of medication order (day and month of the year) and levels of medication ordering errors were evaluated. The author concluded that medication ordering error by trainees remains common, despite electronic medical records. Further supervision and resident training may be necessary to minimize the incidence of prescribing errors [12].

On the whole, the entry of CPOE in most hospitals leads to improved quality of health care service and reduces ADE. Nevertheless, medication error documentation has been increased in large tertiary hospitals, causing documentation fatigue and inappropriate reporting. This is due to either inadequate prescriber training or order entry problems leading to such errors. This study aimed to analyze interventions made by pharmacists during the verification of computerized physician order entries and to determine the association between prescribers’ level and type of prescribing errors for hospitalized patients at the national guard hospital, Jeddah.

## 2. Materials and Methods

### 2.1. Place of Study

The current study was conducted at King Abdulaziz Medical City (KAMC) hospital in Jeddah in 2020 in an inpatients setting from 1 January 2020 to 31 March 2020.

### 2.2. Study Design, Setting, and Patient Population

This cross-sectional, observational study was approved by the institutional review board at King Abdullah International Medical Research Center (KAIMRC-RJ20/069/J). The data collection was conducted over three months starting from 1 January 2020 to 31 March 2020 by using a validated data collecting sheet which was evaluated by two senior pharmacists in the hospital pharmacy field.

### 2.3. Therapeutic Interventions Documentation (TID) Reporting at KAMC-J

The CPOE system in KAMC mandated the pharmacist to report all interventions taken on any order prescribed by the physicians. TID is an online reporting system within the hospital CPOE system “Bestcare^®^”, enabling the healthcare professional to report all modifications performed on the prescriber orders. Each report contained the date and time of interventions, patient details, medication, staff, type of intervention involved and prescriber decision and information. Each intervention in the study sample during the study period was evaluated and analyzed to determine the intervention classification based on the predetermined definitions (Appendix A) [15]. The intervention was considered appropriate when the documented intervention by the pharmacist matched with the real cause of prescribing error modification.

### 2.4. Sample Size

A single population proportion formula was used to calculate the sample size [16]:

X = Z_α/2_^2^ × p × (1 − p)/MOE^2.^

Z_α/2_ is the critical value of the normal distribution at α/2 (e.g., for a confidence level of 95%, α is 0.05 and the critical value is 1.96).

MOE is the margin of error; p is the sample proportion.

X = (1.96)^2^ × 0.5 × (1 − 0.5)/(0.02)^2^

X = 2401.

### 2.5. Sampling Technique

A stratified sampling technique was used to determine the sample of this study. The selected sample was matched proportionally to each main intervention and sub-intervention in the primary data. The TID sample based on each main intervention and sub-intervention was selected from the original TID list using a simple random sampling technique in Microsoft Excel to generate random numbers.

### 2.6. Groups

The medication was categorized according to the WHO Anatomical Therapeutic Chemical (ATC) classification [17]. Every inappropriate intervention was corrected and included in the final analysis. Interventions account for ≥ 3% of the total interventions handled as a separate group. In contrast, the others were merged under a new group named Others Intervention. This new classification produced six unique groups, representing 93.4% of the total interventions, while the merged ones accounted for 6.6%. On the other hand, the prescriber level was divided into four categories—attending physician (consultant, associate consultant, and assistant consultant), specialist (staff physician, fellow), senior physician (resident-R5, resident-R4) and junior physician (all the remaining).

### 2.7. Primary Endpoints

- To find out the frequency and type of interventions made by pharmacists during verification of CPOE orders.
- To evaluate the appropriate documentation of interventions made by pharmacists on prescribing process.

### 2.8. Secondary Endpoints

- To find the association between prescribers’ level and types of prescribing error.
- To evaluate the association of prescriber ordering time with types of prescribing error.
- To describe the variation between different specialties in all types of error.

### 2.9. Statistical Analysis

All data were analyzed using the Statistical Package for the Social Sciences (SPSS) version 26.0 (SPSS Inc., Chicago, IL, USA). Categorical data were expressed as frequencies and percentages. The comparisons between the two groups were performed using contingency table analysis with a χ^2^ test and post hoc Bonferroni correction for multiple comparisons, *p*-value < 0.05 was considered significant.

## 3. Results

Between 1 January 2020 and 31 March 2020, a total of 14,144 interventions were carried out by 52 pharmacists. Within the same period, the total number of inpatient orders was approximately 155,649, which were entered into the CPOE system by different physician level (ranging from consultant to resident-1 (R1)). The prevalence of prescribing order entry errors was 9.1% of the total orders.

### 3.1. Frequency and Type of Interventions Done by Pharmacists during Verification of CPOE Orders

**Figure 1:**
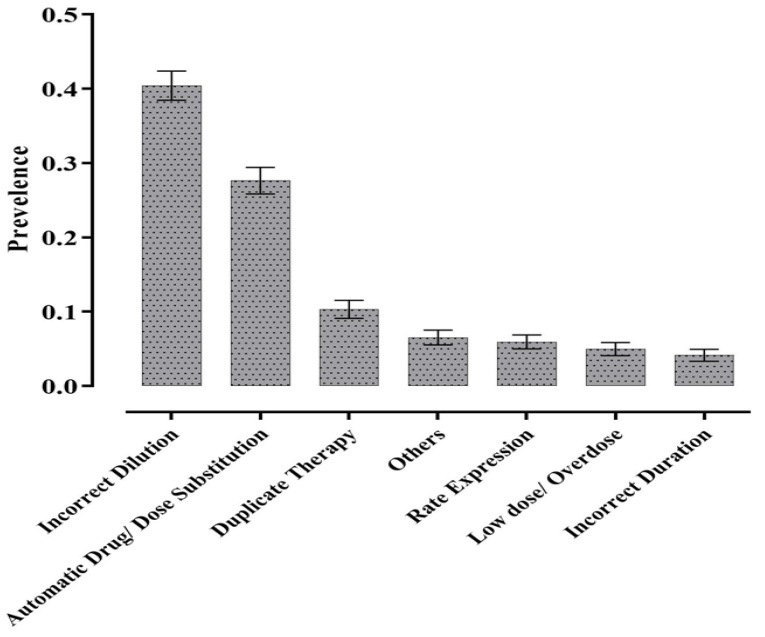
The proportion and the 95% confidence interval (CI) of therapeutic interventions documented by pharmacists for prescribing errors during verification of CPOE orders. *n* = 2405.

The study sample encompassed 2405 interventions documented by 52 different pharmacists during the study period. The most commonly identifiable type of intervention was incorrect dilution (*n* = 972, 40.2%) followed by dose substitution and duplicate therapy (*n* = 665, 27.7%), and (*n* = 248, 10.3%) respectively (Figure 1).

**Figure 2:**
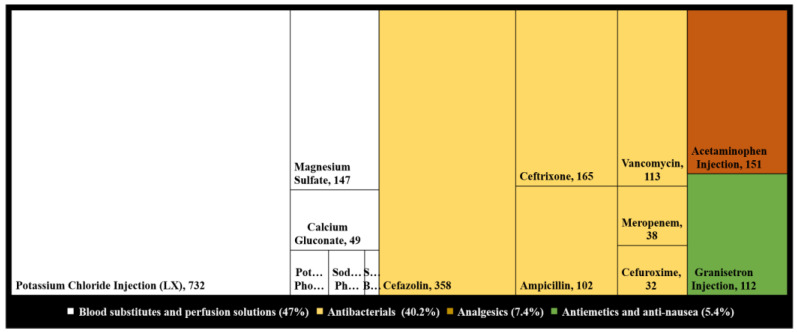
Medications associated with a high percentage of interventions based on ATC classification represented with a treemap; medications have less than 20% of interventions are shown in acronyms; *n* = 2087.

Moreover, the drug category associated with a high percentage of interventions based on ATC classification was blood substitutes and perfusion solutions (*n* = 981, 41%), followed by antibacterial for systemic use and analgesics (*n* = 839, 35%), and (*n* = 154, 6.4%), respectively. The main therapeutic subgroups frequently reported within blood substitutes and perfusion solutions were electrolyte solutions; potassium chloride 74.6%, magnesium sulfate 15% and calcium gluconate 5%. Also, cefazolin, ceftriaxone and vancomycin were responsible for 40%, 20%, and 15%, respectively, of the total interventions in the antibacterial category, as shown in (Figure 2).

### 3.2. Appropriateness of the Interventions Were Documented by Pharmacists on Prescribing Process

**Figure 3:**
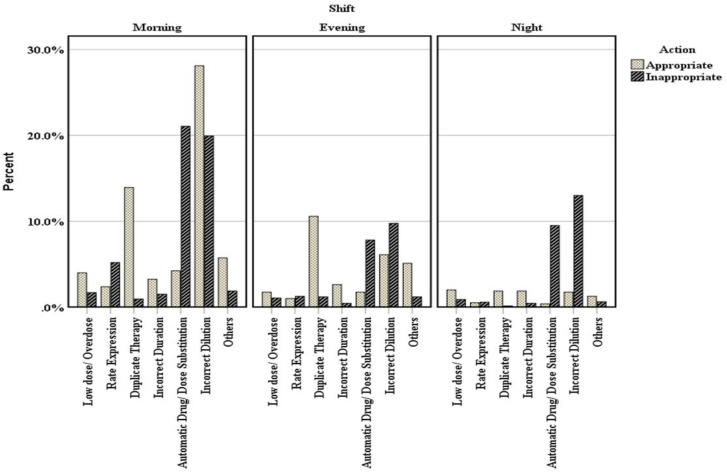
Distribution of the interventions were performed by pharmacists against different types of prescribing errors in daily duty time based on the source of the appropriateness of intervention was taken.

Among 2405 documented interventions, (1600 (66.5%)) interventions were documented inappropriately while the appropriate documented interventions were (804 (33.4%)). Moreover, 52% of the inappropriate interventions were reported in the morning duty. Also, the documented interventions during evening and night duty were lower than that of the morning duty (23% and 25%, respectively (Figure 3)). Furthermore, out of 804 appropriate interventions, 289 (36%) come from incorrect dilution, while the duplicate therapy represented 26% of the total interventions. On the contrary, most inappropriate interventions were documented within the incorrect dilution category (*n* = 683, 43%). Furthermore, there was an increase in the odds of appropriate documentation among pharmacists working in the morning and evening duty compared with night duty (OR = 3.095, 95% CI = 2.36–4.04), (OR = 3.33, 95% CI = 2.48–4.4), respectively.

### 3.3. Association between Prescribers’ Level and Types of Prescribing Errors

**Figure 4:**
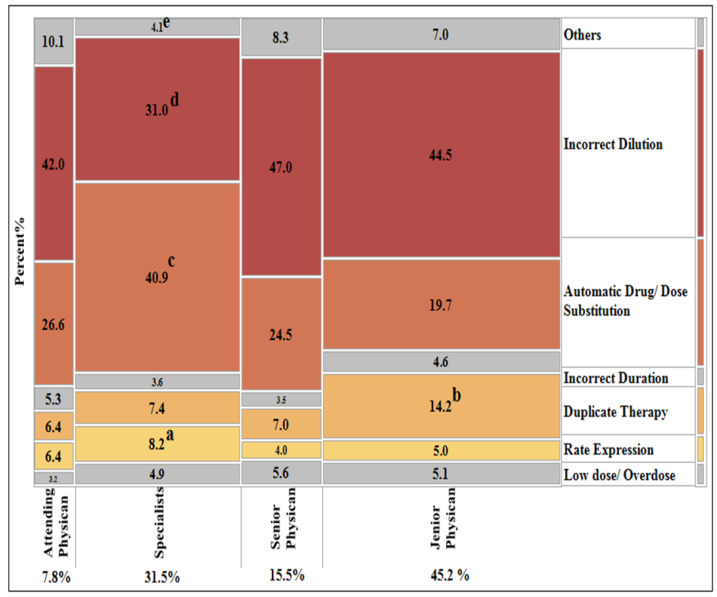
Mosaic plot representing the distribution of pharmacist interventions for prescribing errors depending on the prescribers’ level. **^a^** *p* < 0.05 compared with junior physician; **^b–d^** *p* < 0.05 compared with all other prescribers’ level; **^e^** *p* < 0.05 compared with senior and attending physician; by post hoc Chi-square tests (Bonferroni adjustment *p*-value = 0.00357).

The number of junior physician orders that required pharmacist intervention was higher than other prescribers (45.2%) followed by specialist and senior physicians (31.4% and 15.5%, respectively). A Pearson’s chi-square test of association revealed a statistically significant relationship (*X*^2^(18, *n* = 2406) = 155.07, *p* < 0.05). Post-hoc tests were done to determine which pairs of prescribers were associated with a high percentage of prescribing errors, which triggered different interventions, as shown in (Figure 4).

### 3.4. Association of Prescriber Ordering Time with the Type of Prescribing Errors

Prescriber ordering time and types of prescribing errors were shown to have a significant association, *X*^2^ (12, *n* = 2405) = 105.84, *p* < 0.05. A post-hoc z-test on the adjusted residuals with Bonferroni correction revealed that for; rate expression, duplicate therapy, automatic drug/dose substitution, and others type of errors there was a significant difference among the different shifts, *p* < 0.05 (Table 1).

### 3.5. The Variation between Different Specialties in Different Types of Prescribing Errors

**Figure 5:**
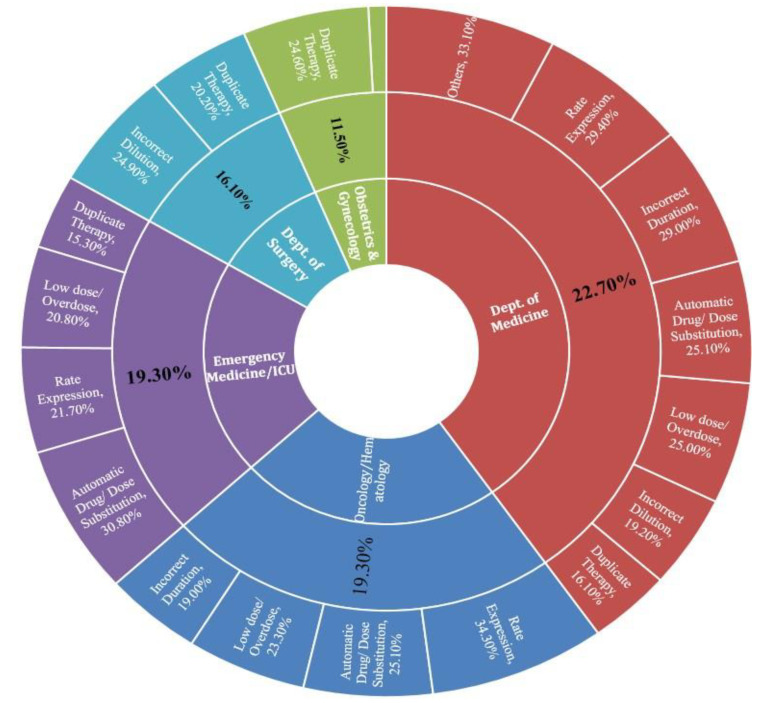
Distribution of prescribing errors among different specialties.

Analysis of prescribing errors rate in various specialties showed that the highest present of error in all types of errors was entered by internal medicine physicians, which represent 22.7%, followed by oncology and ER physicians, which displayed an equal number of errors rate of 19.3%. Of the reported rate expression interventions, 34.3% were entered by oncology physicians and 29.4% by internal medicine physicians. On the other hand, 24.6% of the duplicate orders that required interventions were prescribed by Obstetrics and Gynecology. Besides that, a large number of reported interventions due to dilutional errors (24.9%) were requested by surgical doctors (Figure 5).

## 4. Discussion

Our study highlights the importance of pharmacist TID. We found that pharmacists play a critical role in correcting and adjusting prescribers’ wrong orders that would have jeopardized patient safety if they had reached them. The documentation of prescribing error will have a positive impact on the whole organization and will improve quality and patient safety. Similar results have been reported in different studies [18,19,20].

In the current study, the prevalence of medication prescribing errors was estimated to be 9.1%. Our results showed a concerningly high prevalence of medication prescribing errors compared to other studies. Honey et al. 2015 reported a prescribing error rate for all training programs of 5.88% in 2941 prescriptions [14]. Another study has shown an incident rate of 3.9% at a tertiary care academic medical center over a four year period [12]. Similarly, Ryan C et al. reported an overall error rate of 7.5% of the prescribed medications. The author concludes that the workload and interruptions from other staff were reported as the leading causes of errors [21]. The lack of proper training of prescribers could explain one reason for this abnormally high percentage. The other reason is that the CPOE order entry module can be user unfriendly and contains many entry options for each medication. These disorganized options may drive the prescriber to choose an incorrect drug, diluent, or rate from the list of drugs proposed by the CPOE system.

The most common type of prescribing error was a dilutional error. This type of error represents around 40.4% of the total interventions made by the pharmacist. Many studies have proven that dilutional error is a common problem in practice. Mendes JR et al. 2017 demonstrated that drug dilution error was detected below the manufacturer’s recommended volume in the São Paulo hospital’s emergency department in 1.6% of the total errors [22].

Automatic drug/dose substitution accounted for 27.7% of the total interventions. Of automatic substitution interventions, 88% were performed on potassium chloride (*n* = 577) out of 655 interventions. This intervention was primarily affected by the regional pharmacy and therapeutics committee’s decision, which approved the automatic substitution of any order of potassium chloride 40meq in 400 mL and 30meq in 300 mL to 35meq in 350 mL without referring to the doctor. As a result of these changes, interventions of automatic drug substitution have jumped to a high percentage due to shifting the correction process to the pharmacist instead of training the prescriber on the new situation.

Moreover, this study demonstrated a high percentage of inappropriate TID documentation—66.5% of the total interventions. A large number of irrelevant intervention documentation might be considered an indicator of intervention documentation *fatigue*, which has not been described before in pharmacy intervention studies. Intervention documentation *fatigue* can be explained by the high incident of entry error, as mentioned earlier. Every 10 min, the pharmacist will receive an order that requires intervention. Assuming the average time for each documentation is 1.5 min, the pharmacist will spend an average of four hours daily on correcting wrongly entered orders. The proper training of the prescriber and a change in the CPOE order entry will lead to a significant reduction in the number of improper documentations as well as to improve the quality of reporting.

The current work showed that the junior physician was highly vulnerable to making entry errors compared to other physicians’ levels. The same result has been reported by Garber et al., who found that the number of prescribing entry errors increased from R4 to R2 [12]. Adding to this, Honey et al. demonstrated that medical training programs could significantly affect residents’ prescribing habits and decrease medication errors [14].

This study has several limitations; it is cross-sectional in nature and the convergence upon prescribers within a single institution restricts the result’s generalizability. In addition to that, analyzing the most predominant interventions, which represent 93.4%, may cause neglect of the interventions with low incidence and considered highly significant. Further to this, all interventions were detected and reported by pharmacists before dispensing and did not include the errors that reached the patient and caused harm due to detection failure.

Finally, launching an extensive training program for all prescribers and modifying the medication order entry option to contain fewer selection options in the COPE system will improve the TID documentation process, and decrease order entry error and TID fatigue. In the same manner, the encouragement of pharmacists to select the right reason for TID will lead to overcoming inappropriate reporting.

## 5. Conclusions

This study showed that routine pharmacist review of inpatient drug therapy is a crucial part of the quality use of medicines with a significant potential to reduce drug related problems. However, identifying and correcting prescriber errors are major challenges faced by inpatient pharmacists. This problem should be addressed and corrected by conducting an extensive training program for all prescribers, modifying the CPOE system to become more friendly, and limiting the entry options to what the pharmacy prepares. Finally, intervention documentation fatigue is a real issue that might be under-reported. Addressing this point in future studies would be of great value.

## Figures and Tables

**Table 1:** Association of prescriber ordering time with type of errors.

		Shift			Total
		Morning	Evening	Night	
Low dose/Overdose	Count	59 **^a^**	31 ^a^	30 ^a^	120
Rate Expression	Count	102 **^a^**	28 ^a,b^	13 ^b^	143
Duplicate Therapy	Count	127 **^a^**	104 ^b^	17 ^c^	248
Incorrect Duration	Count	50 **^a^**	28 ^a^	22 ^a^	100
Automatic Drug/Dose Substitution	Count	371 **^a,b^**	139 ^b^	155 ^a^	665
Incorrect Dilution	Count	545 **^a^**	205 ^b^	222 ^a^	972
Others	Count	77 **^a^**	60 ^a,b^	20 ^a^	157

Each superscript letter denotes a subset of Shift categories whose column proportions do not differ significantly from each other at the 0.05 level; by *z*-tests for independent proportions (Bonferroni adjustment *p*-value = 0.00357).

## Data Availability

Not applicable.

## Appendix A

**Table A1:** Definitions and criteria used in identification of type of interventions [15].

Therapeutic duplication	This refers to the simultaneous prescription of two or more drugs with the same active substance or the same pharmacological action.
Incorrect Dilution	Dilution of medication in a diluent that is different or in smaller/larger volume than what is recommended by the hospital standard manual.
Automatic Drug/Dose Substitution	The practice of replacing a patient’s prescription drugs with chemically different drugs that are expected to have the same clinical effect.
Decimal point	Is any error when the decimal point is misplaced, can lead to misinterpretation of prescriptions.
Contraindication	A contraindication is a specific situation in which a drug, should not be used because it may be harmful to the person
Drug interaction	A change in the way a drug acts in the body when taken with certain other drugs.
History Taking	Information is gained by pharmacist through CPOE to answer specific questions and to obtain information useful in medication selection and to connect patient diagnosis with selected treatment.
Incorrect dosage form	Inappropriate use of a specific dosage form, or the failure to specify the correct dosage form when more than 1 dose form is commonly available
Incorrect Duration	Duration errors occur when medication is received for a longer or shorter period of time than prescribed.
Allergy	Dispensing a drug that the patient has an allergy often due to failure to communicate with the patient, inappropriate chart review, inaccurate charting, or lack of technologic interface.
Incorrect frequency	When the prescribed frequency of a medicine is different from current evidenced based treatment guidelines
Incorrect Route	When a route not intended by the prescriber is written by him/her on the medication order.
Low dose/Overdose	The inappropriate low or high dose is ordered other than what was recommended in the evidenced based treatment guidelines.
Rate Expression	Most often occurs with medications given as IV push or infusions when the prescriber selects inappropriate infusion time.
Renal/hepatic dose adjustment	It occurs when the prescriber is not considering renal or liver failure, and he ordered full dose for the Patients with renal and liver dysfunction without adjustment.
Editing Arabic Instruction	It is defined as an intervention when the pharmacist adds extra instruction and precautions when necessary when dispensing the drugs.
Unit of Measure	when the wrong strength is written in terms of units e.g., mg written instead of micrograms
